# Study on the Environmental Impact and Benefits of Incorporating Humus Composites in Anaerobic Co-Digestion Treatment

**DOI:** 10.3390/toxics12050360

**Published:** 2024-05-13

**Authors:** Ke Zhao, Qiang Wei, Mingxuan Bai, Mengnan Shen

**Affiliations:** Key Laboratory of Songliao Aquatic Environment, Ministry of Education, Jilin Jianzhu University, 5088 Xincheng Street, Changchun 130118, China; zhaoke@jlju.edu.cn (K.Z.); weiqiang@student.jlju.edu.cn (Q.W.); baimingxuan@student.jlju.edu.cn (M.B.)

**Keywords:** humus composites, anaerobic co-digestion, life cycle assessment, environmental impact, benefit evaluation

## Abstract

This study evaluated the environmental impact and overall benefits of incorporating humus composites in the anaerobic co-digestion of kitchen waste and residual sludge. The life cycle assessment method was used to quantitatively analyze the environmental impact of the entire anaerobic co-digestion treatment process of waste, including garbage collection, transportation, and final product utilization. Moreover, the comprehensive assessment of the environmental impact, energy-saving and emission-reduction abilities, and economic cost of using humus composites in the anaerobic co-digestion treatment process was conducted using a benefit analysis method. The results showed that the anaerobic co-digestion of kitchen waste and residual sludge significantly contributed to the mitigation of global warming potential (GWP), reaching −19.76 kgCO_2_-eq, but had the least impact on the mitigation of acidification potential (AP), reaching −0.10 kgSO_2_-eq. In addition, the addition of humus composites significantly increased the production of biogas. At a concentration of 5 g/L, the biogas yield of the anaerobic co-digestion process was 70.76 m^3^, which increased by 50.62% compared with the blank group. This amount of biogas replaces ~50.52 kg of standard coal, reducing CO_2_ emissions by 13.74 kg compared with burning the same amount of standard coal. Therefore, the anaerobic co-digestion treatment of kitchen waste and residual sludge brings considerable environmental benefits.

## 1. Introduction

In 2020, China produced ~1.4 × 10^8^ tons [1] of urban food waste, and this amount has consistently increased each year. Owing to severe environmental pollution and scarcity of energy resources, effectively managing kitchen waste has emerged as a critical issue demanding urgent attention. Kitchen waste contains numerous organic compounds that readily decompose through biodegradation. The improper disposal of waste can result in carbon emissions, worsening global warming. The anaerobic treatment of kitchen waste produces methane, a potent greenhouse gas. However, this collected methane can be used as recycled energy to replace fossil fuels, thereby aiding in reducing carbon emissions. This dual benefit has received significant attention in sustainability practices.

Anaerobic co-digestion treatment technology refers to the method of anaerobic digestion treatment of two or more organic wastes at the same time according to the characteristics of different biomass wastes. Several studies have examined the anaerobic co-digestion of kitchen waste and residual sludge to balance the nutrient ratio of the substrate [2], which results in complementary effects and improves gas production efficiency [3,4]. Pan Y et al. investigated the synergistic effect and biodegradation kinetics of residual sludge and kitchen waste in the anaerobic co-digestion process. The results revealed that at a 1:1 ratio of residual sludge-to-kitchen waste, the sludge achieved a significantly high rate of methane recovery, leading to an increase in methane productivity by 4.59 times [5]. Some scholars have studied the hydrolysis performance of the anaerobic co-digestion process of kitchen waste and residual sludge, and the results show that the ratio of 1:1 produces the largest synergistic effect, 38%, and the addition of kitchen waste compensates for C/N, which is conducive to promoting biodegradation [6]. Antony D et al. studied the difference between single digestion and co-digestion at a medium temperature of 35 °C. The results showed that the maximum methane yield was 43.6% when the optimal ratio of kitchen waste and residual sludge was 1:1 [7].

Additionally, Guo et al. investigated the energy-saving and emission-reduction capacities of the anaerobic co-digestion treatment for residual sludge and kitchen waste. The findings indicated that compared with landfills, the anaerobic co-digestion treatment of 105 t/day of waste could reduce CO_2_ emissions by 4.26 t, leading to significant environmental benefits [8]. The anaerobic co-digestion treatment of kitchen waste and residual sludge saves infrastructure investment, significantly enhances the conversion of biomass into energy, improves energy and nutrient recovery rates, and reduces greenhouse gas emissions [9,10]. With the continuous improvement of environmental management standards, anaerobic co-digestion treatment technology may emerge as an effective strategy for controlling actual pollution from sewage treatment plants and kitchen waste management departments in the future [4]. Therefore, incorporating comprehensive assessments of environmental benefits into the process evaluation system is crucial.

In this study, the anaerobic co-digestion treatment project of kitchen waste and residual sludge with humus composites was selected as a case study. The life cycle assessment method was used to quantitatively analyze the environmental impact of the entire waste treatment process, including garbage collection, transportation, and final product utilization. Additionally, the energy-saving and emission-reduction capabilities and economic costs associated with using humus composites in anaerobic co-digestion treatment were investigated. A comprehensive evaluation of the environmental impact and economic benefits of the waste treatment process provided valuable insights for optimizing waste recycling and sewage treatment processes. This optimization resulted in reduced operating costs for waste recycling stations and sewage treatment plants, thereby promoting the sustainable development of urban organic solid waste treatment and sewage sludge treatment. Finally, this approach achieved efficient resource recycling and environmental protection goals. This study provides valuable guidance for achieving a balance between low-carbon emissions, environmentally friendly practices, and sustainable economic development.

## 2. Materials and Methods

### 2.1. Laboratory Research

The kitchen waste, sourced from the school canteen, mainly comprised starch (from rice and steamed bread), cellulose (from fruits and vegetables), and protein (from chicken and fish), with a mass ratio of 2:1:1. Subsequently, the waste was processed into a pulp using a crusher and then packed, sealed, frozen, and stored at −20 °C. When needed for use, the waste pulp was thawed at room temperature, and its content was adjusted through the addition of tap water. The unused sludge was then stored in the refrigerator at −4 °C for future use. The remaining sludge used in the experiment was obtained from the sludge-thickening tank of the municipal sewage treatment plant [11]. Kitchen waste and residual sludge are wet bases with moisture contents of 85% and 95%, respectively. The experiment was performed in serum bottles with a total volume of 500 mL, with each anaerobic digester operating at a working volume of 400 mL. The biochemical methane potential was tested under constant temperature conditions of 35.0 ± 1 °C and a stirring speed of 500 rpm/min. Biogas production was regularly monitored throughout the experiment.

**Figure 1:**
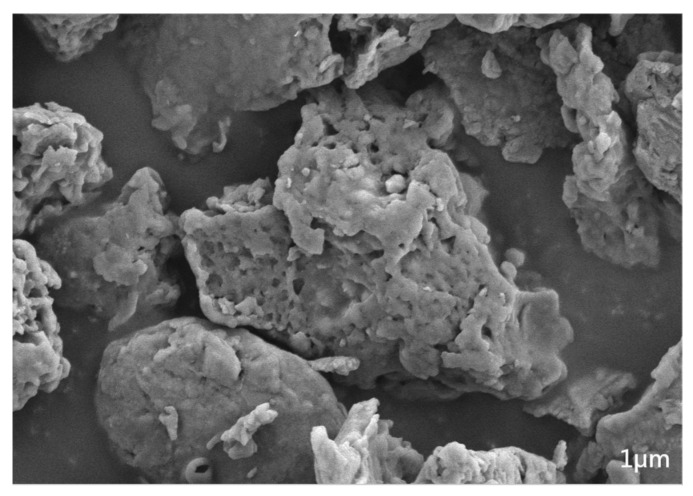
Scanning electron microscope picture of humus composites.

The humus composites, which came from a company in Changchun City, is a yellow powder solid with a little earthy taste, and the organic matter content is 82.14%. The surface of the humus composites has a porous and loose structure, as shown in Figure 1. This structure is likely to provide better support for the adhesion of electron-giving bacteria and electron-accepting methanogenic bacteria to the material for electron exchange, enhance the ability of the humus composite biological filler as an electronic medium or shuttle, and promote the gas production efficiency of anaerobic co-digestion. In addition, humus composites contain metal elements such as Fe and Zn, which play a key role in promoting the anaerobic digestion process in related studies [12]. The element content of humus composites is shown in Supplemental Material Table S1. Detailed information regarding the properties and data of relevant raw materials can be found in Supplementary Materials Tables S2 and S3.

### 2.2. Life Cycle Assessment

In accordance with extensive investigations and the research of numerous scholars, this study selected several types of environmental impacts, including global warming potential (GWP), acidification potential (AP), eutrophication potential (EP), and human health toxicity environmental potential (HTP). These factors were used to evaluate the impact of the anaerobic co-digestion process of kitchen waste and residual sludge on different aspects [13,14]. Information on the potential values of these impact factors can be found in Tables S4–S6.

(1) $$GWP=\sum_{i=1}^{n}\delta_{i}\times D_{GWP},$$

(2) $$AP=\sum_{i=1}^{n}\delta_{i}\times D_{AP},$$

(3) $$EP=\sum_{i=1}^{n}\delta_{i}\times D_{EP},$$

(4) $$HTP=\sum_{i=1}^{n}\delta_{i}\times D_{HTP},$$

where n represents the type of pollutant discharged; $\delta_{i}$ denotes the equivalent coefficient of pollutants, i; $D_{GWP}$ signifies the emissions of greenhouse gas, i; $D_{AP}$ indicates the emissions of acidifying gas, i; $D_{EP}$ represents the emissions of eutrophication pollutant, i; and $D_{HTP}$ denotes the emissions of pollutants with a toxic impact on humans, i.

### 2.3. Economic Cost Assessment Method

A practical approach for assessing the economic viability of using additives was to compare whether an increase in gas production due to the additive outweighed its cost. The economic cost assessment did not include the expenses related to transportation, treatment equipment, and plant construction during anaerobic co-digestion. Because transportation is the necessary cost of any food waste treatment method, and the collection and transportation process has less impact than the treatment and utilization, some treatment equipment and plant construction can be completed by transforming the existing equipment and facilities of the sewage plant. This is a long-term cost-saving strategy, so the transportation, treatment equipment, and plant construction costs are ignored. The details are as follows:

(5) $$B_{N}\left(RMB\right)=B_{C}-B_{A},$$

(6) $$B_{C}\left(RMB\right)=U_{B}\times P_{B},$$

(7) $$B_{A}\;\left(RMB\right)=U_{A}\times P_{A},$$

where $B_{N}$ represents net income after the addition of additives (RMB), $B_{C}$ denotes biogas revenue (RMB), $B_{A}$ indicates the cost of additives (RMB), $U_{B}$ signifies the production of biogas (m^3^), $P_{B}$ represents the unit price of biogas sales (3.97 RMB/m^3^), $U_{A}$ indicates the dosage of additives (kg), and $P_{A}$ represents the unit price of additives (197.00 RMB/kg).

### 2.4. Energy Conservation and Emission-Reduction Assessment Method

Gao et al. [15] proposed methods for calculating the replacement of standard coal with biogas of equivalent heat and for evaluating CO_2_ emissions from the combustion of biogas and coal. These methods are presented in the following formula. Additionally, the correlation coefficient used in these calculations was derived from the research conducted by Ren et al. [16].

(8) $$Q_{C}\left(m^{3}\right)=Q_{B}\times E,$$

(9) $$C_{B}\left(t\right)=3.67\times B\times q_{B}\times E_{B}\times 99\%,$$

(10) $$C_{C}=3.67\times C\times q_{C}\times E_{C}\times 94\%,$$

where $Q_{B}$ represents methane production (m^3^), $Q_{C}$ denotes standard coal (kg), E indicates the conversion factor of biogas and standard coal (with a value of 0.714 kg/m^3^), *C_B_* represents CO_2_ emissions from biogas combustion (t), 3.67 signifies the relative molecular weight ratio of CO_2_ to C, B signifies methane consumption (m^3^), $q_{B}$ indicates the calorific value (0.209 TJ/10^4^ m^3^) of biogas, $E_{B}$ represents the carbon content per unit calorific value of biogas (15.32 t-c/TJ), 99% indicates the carbon oxidation rate of biogas, $C_{C}$ denotes CO_2_ emissions from coal after combustion (t), C represents standard coal energy consumption (kg), $q_{C}$ indicates the calorific value of standard coal (0.0209 TJ/t), $E_{C}$ represents the carbon content per unit calorific value of standard coal (26.37 t-c/TJ), and 94% indicates the carbon oxidation rate of standard coal.

## 3. Results and Discussion

### 3.1. Environmental Impact Assessment of Anaerobic Co-Digestion of Kitchen Waste and Residual Sludge

**Figure 2:**
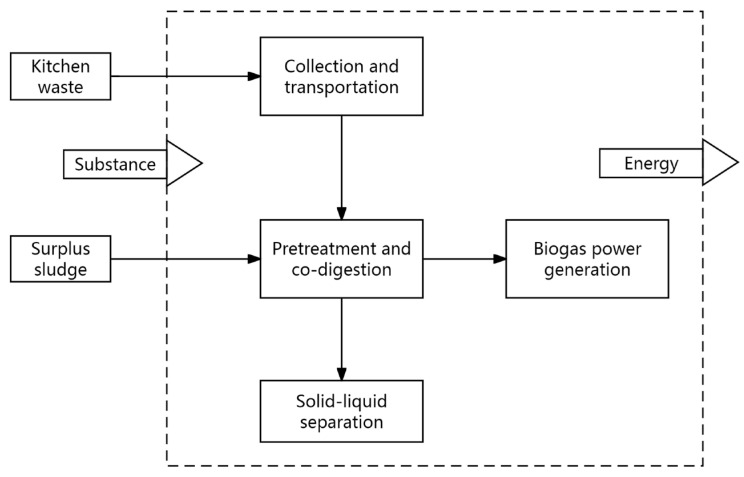
System boundary of anaerobic co-digestion treatment for kitchen waste and sewage sludge.

Life cycle assessment is a tool used to assess environmental impact across different aspects. The steps of this approach involve defining objectives and scope, performing inventory analysis, assessing lifecycle impacts, and interpreting results [17,18]. This study aimed to assess the environmental impact of the entire life cycle of the anaerobic co-digestion treatment process of food waste and residual sludge. Regarding numerous actual engineering data on the anaerobic treatment process, relevant standards, and extensive consultation of the literature, the system boundary diagram was established (Figure 2). To ensure representative and irrelevant life cycle evaluation results, processes such as plant construction, equipment production, use, and scrapping were omitted. This decision was made considering the prolonged service life of the basic equipment of the plant and its minimal average impact on the overall economy and environmental protection. Additionally, non-centralized monitoring processes were excluded from the system’s boundaries [19]. All described operations were confined within these boundaries.

The data used in the inventory analysis were obtained from laboratory research results and engineering case studies described in the relevant literature. The project case processing scale adopted was 150 t/d, and the process route adopted was “pre-separation + medium temperature wet anaerobic digestion + biogas cogeneration + biogas residue composting”, which can be used as a typical case for reference [20]. Predefined processes were selected based on system boundaries, functional units, and geographic locations. Table 1 summarizes the list of each process. (1) Garbage collection and transportation: In this process, the main fuel used was diesel. Market research indicated that a truck carrying a load of 5 t consumed ~0.2 L of fuel per km. Assuming an average collection and transportation distance of 100 km, then each ton of kitchen waste collection and transportation process would require 4 L of diesel. At a diesel density of 0.84 g/cm^3^, the diesel consumption for this transportation process was ~3.36 kg. (2) Production of biogas through pretreatment and co-digestion processes: The processing scale was 150 t/day. The pretreatment process operated for 8 h/day, with the total power of the equipment at 280.75 kW and a power consumption of ~15 kWh/t. The co-digestion process operated for 24 h per day, with the total power of the equipment at 49.6 kW and a power consumption of ~8 kWh/t. The total power consumption for both processes amounted to 23 kWh/t. The odor treatment capacity was 93,000 Nm^3^/h, and the main components of the odor emission were NH_3_ and H_2_S, with emission concentrations of 7 mg/m^3^ for NH_3_ and 0.3 mg/m^3^ for H_2_S. (3) Solid–liquid separation and biogas slurry treatment: The solid–liquid separation unit operated for 8 h/day, with the total power of the equipment at 140 kW and power consumption of ~7.5 kWh/t. The power consumption for biogas slurry treatment was not calculated because the slurry was returned to the sewage treatment unit of the sewage plant. (4) Biogas power generation: After basic treatment, the biogas was sent to the generator set for power generation. The emission coefficient of flue gas pollution from the generator set was determined based on the research conducted by Amon et al. [21].

**Figure 3:**
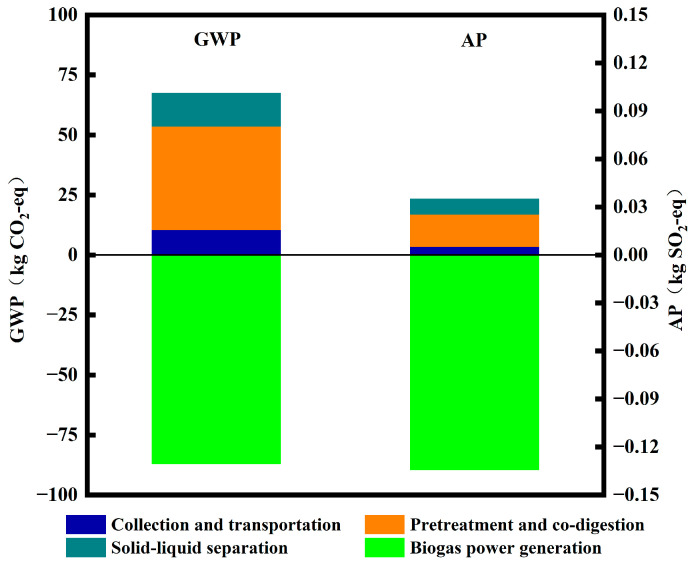
Effects of GWP and AP at different stages of the treatment process.

According to the collected data, each environmental impact category was converted to the equivalent of the same characteristic pollutant using the equivalence method to calculate the environmental impact at each stage of the life cycle. With reference to the studies by several scholars, this study selected four types of environmental impacts that significantly influenced the results [22,23]. The effects of global warming and acidification are shown in Figure 3. In summary, the anaerobic co-digestion of kitchen waste and residual sludge significantly contributed to mitigating GWP. Processes such as garbage collection and transport, pretreatment, co-digestion, and solid–liquid separation had a positive impact on GWP. However, the recovery and utilization of biogas resulted in a negative GWP value for biogas power generation. Overall, the combination of these processes resulted in a GWP of −19.76 kgCO_2_-eq. Similar results were obtained in the environmental impact assessment of anaerobic digestion of kitchen waste, with a GWP of −0.855 kgCO_2_-eq [24]. This strongly suggests that anaerobic co-digestion could contribute to mitigating global warming potential. Moreover, the anaerobic co-digestion treatment of kitchen waste and residual sludge had a minimal effect on AP, with an overall AP of −0.10 kgSO_2_-eq. This indicates that the anaerobic co-digestion system efficiently uses kitchen waste and residual sludge to recover energy, thereby mitigating environmental loads. Additionally, this technology can reduce the environmental impact associated with climate change and fossil energy consumption, thereby promoting the achievement of carbon dioxide “zero emissions” and contributing to carbon neutrality. China, as the world’s largest greenhouse gas emitter, has made addressing global warming a top priority. The United Nations Framework Convention on Climate Change urges countries to implement measures for reducing emissions [17]. The extensive use of fossil fuels has significantly influenced the environment, resulting in the release of key pollutants, such as sulfur dioxide, suspended particles, nitrogen oxides, and carbon dioxide, that have led to severe environmental pollution [25]. Therefore, a fundamental shift is required in the methods involved in the production, transportation, and consumption of energy [26]. Furthermore, pathways for waste recycling must be established to effectively reduce greenhouse gas emissions.

**Figure 4:**
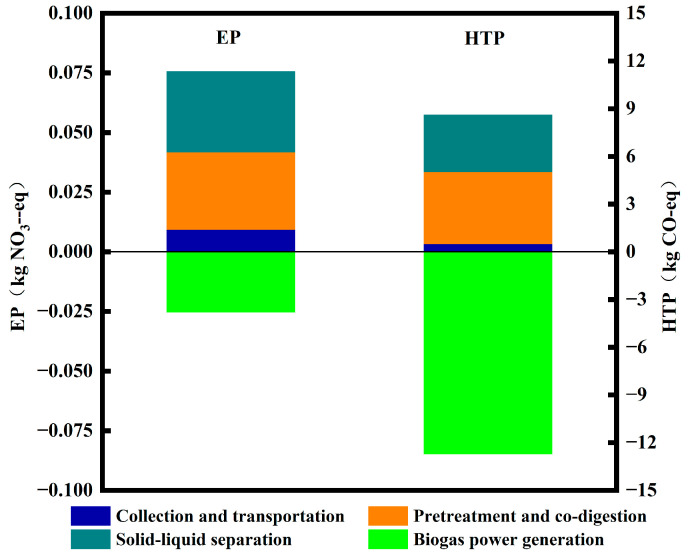
Effects of EP and HTP at different stages of the treatment process.

The effects of EP and HTP on the anaerobic co-digestion system of kitchen waste and residual sludge are shown in Figure 4. The life cycle EP of the anaerobic co-digestion system was −0.05 kg NO_3_-eq, and HTP was −4.11 kg CO-eq. Similar results for EP were reported in related studies. Oldfield et al. reported an EP of −0.003 kgNO_3_-eq, Slorach et al. estimated EP to be 1.24 kgNO_3_-eq, and Fei et al. suggested EP to be 0.19 kgNO_3_-eq [14,27]. However, a direct comparison of these results with those reported by relevant scholars [28] was challenging owing to differences in system boundaries, assumptions, and methods used for life cycle impact assessments. Overall, anaerobic digestion treatment technology had the lowest impact on global warming compared with composting, incineration, and landfilling. Composting significantly contributed to eutrophication while landfilling had the highest environmental impacts, including AP, GWP, EP, and HTP [29,30]. Even on a relatively small scale, anaerobic digestion treatment technology has contributed to energy conservation and emission reduction with certain environmental significance [31]. However, the impact of anaerobic digestion treatment technology on the environment is limited by various factors, such as the biogas production potential of the substrate, process scale, and technical route. Further improvements in the treatment technology can enhance its positive impact on the environment in the future.

### 3.2. Evaluation of the Economic Impact of Incorporating Humus Composites in Anaerobic Co-Digestion Treatment

**Figure 5:**
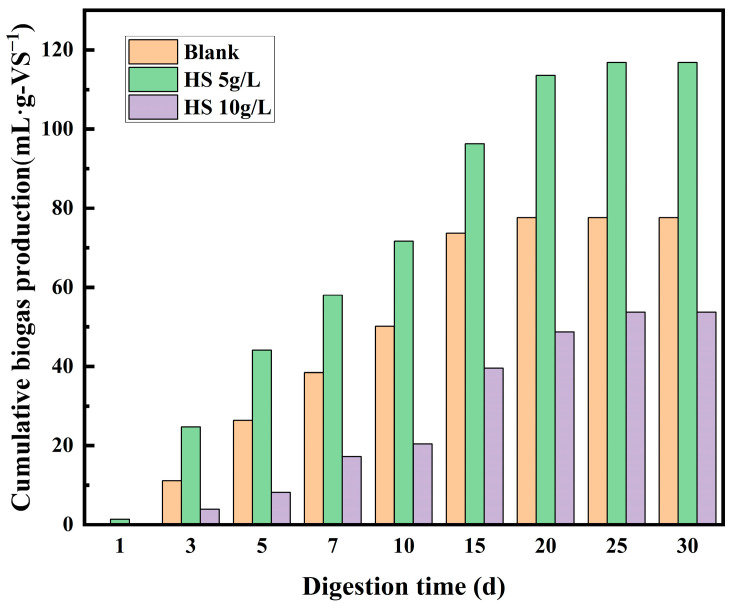
Changes of cumulative biogas production with digestion time.

With its unique performance, humus composites contribute to improving the performance of anaerobic co-digestion and recovery of biogas from kitchen waste and residual sludge, thereby reducing the impact of organic solid waste on the environment. Laboratory experiment results show that 5g/L is the best dosage, and the co-digestion treatment with 5g/L will produce more biogas, as shown in Figure 5. Biogas from waste recovery produces less CO_2_ when burned than the same amount of coal, helping to reduce CO_2_ emissions. This improvement contributed to enhanced energy conservation and emission-reduction capabilities, thereby reducing the environmental impact of waste treatment and yielding good environmental benefits.

In the anaerobic co-digestion treatment system of kitchen waste and residual sludge, Table 2 presents the economic cost analysis results of incorporating humus composites. Without the addition of humus composites, the anaerobic co-digestion of 1 t of kitchen waste and residual sludge can yield a net income of RMB 186.51 generated from the production of biogas. The cost associated with the addition of humus composites at a standard concentration of 5 g/L was RMB 985.00 per ton, leading to a reduction in net income. However, the addition of humus composites can shorten digestion time and significantly increase biogas production. The addition of humus composites at a standard concentration of 5 g/L can result in the production of 70.76 m^3^ biogas, representing a 50.62% increase in biogas production and enhancing energy recovery efficiency. Some scholars have increased the gas production of anaerobic digestion through the addition of biochar, bentonite, and zero-valent irons [1,32,33]. Although various additives can improve gas production efficiency to a certain extent, their cost can affect economic benefits. Gao [34] found that the addition of 7.5% magnet powder to the anaerobic treatment of 1 t of kitchen waste incurred a cost of RMB 543.75 per ton, resulting in only a 36.61% increase in biogas production. In contrast, humus composites exhibited more advantages in enhancing biogas production efficiency. With the advancement of treatment technology and increasing treatment scale in the future, achieving a balance between treatment costs and benefits will be crucial.

### 3.3. Assessment of Energy Conservation and Emission-Reduction Effects in Anaerobic Co-Digestion Treatment with the Addition of Humus Composites

Additives were used to enhance biogas production in the anaerobic co-digestion treatment process and mainly exhibited energy-saving and emission-reduction effects in two aspects. First, biogas can replace coal as an energy source. Second, compared with coal, the use of biogas can effectively reduce the emissions of greenhouse gases, particularly CO_2_ [35]. The energy-saving and emission-reduction capacities of biogas can be expressed by comparing the amount of CO_2_ released during biogas combustion with that released from coal combustion, which was replaced by biogas.

In the anaerobic co-digestion treatment system of 1t of kitchen waste and residual sludge containing total solids, the analysis of energy-saving and emission-reduction capacities of biogas produced by humus composites is shown in Table 3. Without the addition of humus composites, the anaerobic co-digestion of kitchen waste and residual sludge produced 46.98 m^3^ of biogas, equivalent to ~33.54 kg of standard coal. Compared with the equivalent amount of standard coal, the use of biogas resulted in a reduction in total CO_2_ emissions by 9.12 kg. Under the same conditions of anaerobic treatment, the addition of humus composites at a standard concentration of 5 g/L produced 70.76 m^3^ of biogas, replacing ~50.52 kg of standard coal. This substitution resulted in a reduction in CO_2_ emissions by 13.74 kg compared with the combustion of an equivalent amount of standard coal. These findings indicate that the addition of humus composites to the anaerobic co-digestion treatment system of kitchen waste and residual sludge can significantly reduce CO_2_ emissions, improve energy-saving and emission-reduction capabilities, and yield considerable environmental benefits for the management of organic solid waste. Guo et al. compared the energy-saving and emission-reduction capabilities of landfill and anaerobic co-digestion [8]. At a sludge treatment capacity of 105 t/d, the landfill generated CO_2_ emissions of 9.66 t, while anaerobic co-digestion produced CO_2_ emissions of only 5.4 t, resulting in a significant reduction of 4.26 t in CO_2_ emissions. The anaerobic co-digestion of kitchen waste and residual sludge can significantly improve energy conservation and emission-reduction capacities, leading to notable environmental benefits. Moreover, elucidating the positive social impact generated by biogas projects was crucial. For example, in Germany, the anaerobic treatment of organic waste for biogas production has increased the additional income of workers and expanded employment opportunities, thereby positively influencing the labor market [36]. Additionally, compared with other treatment processes, anaerobic processes can reduce carbon emissions and minimize environmental impact. In the future, with the continuous improvement of biogas equipment manufacturing, advancements in biogas technology research and development, and the scaling up of treatment, the overall benefits of the anaerobic co-digestion treatment process will continuously increase.

## 4. Conclusions

This study examined the environmental impact and economic benefits of incorporating humus composites in the anaerobic co-digestion treatment system for kitchen waste and residual sludge. The key findings are summarized as follows: (1) The life cycle assessment of anaerobic co-digestion revealed a GWP and AP of −19.76 kgCO_2_-eq and −0.10 kgSO_2_-eq, respectively, indicating that the overall environmental impact can be reduced. This suggests the environmentally friendly nature of the anaerobic co-digestion process for kitchen waste and residual sludge. (2) The cost of implementing the anaerobic co-digestion treatment with the standard addition of 5 g/L humus composites was RMB 985.00 per ton. Despite the decrease in the net income resulting from the addition of humus composites, it can significantly improve the waste disposal rate and increase biogas production. In the co-digestion treatment system, the addition of humus composites at a concentration of 5 g/L produced 70.76 m^3^ of biogas, replacing ~50.52 kg of standard coal. Consequently, this led to a total reduction of 13.74 kg in CO_2_ emission, which significantly enhanced both energy-saving and emission-reduction capabilities of the system, thereby yielding substantial environmental benefits. These results provide guidelines for optimizing the engineering practices and operational management standards of anaerobic co-digestion treatment technology. This study provides valuable guidance for achieving a balance between low-carbon emissions, environmentally friendly practices, and sustainable economic development.

## Figures and Tables

**Table 1:** Summary of life cycle lists.

Collection and Transportation		Pretreatment and Co-Digestion	
Diesel oil (kg)	3.3600	Pretreatment unit (kWh)	14.9733
CO_2_ (kg)	10.4022	Co-digestion unit (kWh)	7.9360
NO_X_ (kg)	0.0068	NH_3_ (kg)	0.1045
SO_2_ (kg)	0.0003	H_2_S (kg)	0.0045
CO (kg)	0.0040	**Biogas power generation**	
**Solid–liquid separation**		Power export	−87.8526
Separation unit (kWh)	7.4667	CO_2_ (kg)	−101.5802
NH_3_ (kg)	0.0232	NO_X_ (kg)	0.1921
H_2_S (kg)	0.0010	CO (kg)	0.2490

**Table 2:** Economic cost statement.

Treatments	Adding Amount (kg)	Unit Price of Additive (RMB/kg)	Additive Cost (RMB)	Biogas Earnings (RMB)	Net Income (RMB)
Blank	0.00	0.00	0.00	186.51	186.51
Humus composites 5 g/L	5.00	197.00	985.00	280.92	−704.08
Humus composites 10 g/L	10.00	197.00	1970.00	129.10	−1840.90

**Table 3:** Energy conservation and emission-reduction capabilities.

Treatments	1 L Biogas Production (mL/g VS)	1 t Biogas Production (m^3^)	Biogas CO_2_ Emissions (t)	Standard Coal (kg)	Coal CO_2_ Emissions (t)	Emission Reductions (kg)
Blank	77.60	46.98	54.65	33.54	63.78	9.12
Humus composites 5 g/L	116.88	70.76	82.32	50.52	96.06	13.74
Humus composites 10 g/L	53.72	32.52	37.83	23.22	44.15	6.32

## Data Availability

The data presented in this study are available on request from the corresponding author.

## Supplementary Materials

The following supporting information can be downloaded at https://www.mdpi.com/article/10.3390/toxics12050360/s1: Table S1: Raw materials parameter; Table S2: Biogas production; Table S3: Table of emission factor coefficient during diesel fuel used; Table S4: Impact factor potential; TableS5: Standardized factors for various types of environmental impacts; Table S6: Standardized factors for various types of environmental impacts.
