# Supplementary material for: Study on the Environmental Impact and Benefits of Incorporating Humus Composites in Anaerobic Co-Digestion Treatment
Source: Toxics. 2024 May 13;12(5):360. doi: 10.3390/toxics12050360 (PMC11125908; doi:10.3390/toxics12050360)
Supplement: Supplementary file 1 [file toxics-12-00360-s001.zip › toxics-2983405-supplementary.pdf]

Supplementary Data for

Study on the environmental impact and benefits of incorporating humus composites in anaerobic co-digestion treatment

Ke Zhao [1], Qiang Wei [1], Mingxuan Bai [1] and Mengnan Shen [1] *

[1] Key Laboratory of Songliao Aquatic Environment, Ministry of Education, Jilin Jianzhu University, 5088 Xincheng Street, Changchun, 130118, China. Ke Zhao: zhaoke326@126.com; Qiang Wei: weiqiang@student.jlju.edu.cn; Mingxuan Bai: baimingxuan@student.jlju.edu.cn; Mengnan Shen: smn930@aliyun.com

* Correspondence: smn930@aliyun.com; Tel.: 86-431-84566407

TableS1. Elemental content of humus composites.

Parameters	K	Na	Mg	Al	Fe	Mn	Zn	Cu
Contents（mg/g）	13.40	12.10	5.80	3.30	2.50	0.19	0.08	0.01

TableS2. Raw materials parameter.

**Parameters**	**KW**	**SS**	**Inoculum**	**Mix substrate**
pH	4.46 ± 0.05	6.69 ± 0.12	7.94 ± 0.04	7.04 ± 0.08
TS (g/L)	135 ± 3.28	33.96 ± 0.99	20.96 ± 0.31	29.75 ± 0.81
VS (g/L)	127.56 ±3.24	20.55 ± 1.02	9.61 ± 1.28	18.01 ± 0.53
VS/TS (%)	97.75 ± 0.09	60.51 ± 1.22	45.85 ± 0.09	60.54 ± 0.14
SCOD (g/L)	103.99 ± 2.16	0.36 ± 0.02	0.52 ± 0.01	1.75 ± 0.02
TCOD (g/L)	324.75 ± 5.25	31.46 ± 0.89	14.53 ± 1.17	44.74 ± 2.15
NH_4^+-N (mg/L)	4.31 ± 0.04	10.11 ± 0.16	253.92 ± 2.35	214.01 ± 1.82

TableS3. Biogas production (ml/g-VS).

	**Blank**	**HS 5g/L**	**HS 10g/L**
1	0.00	1.39	0.00
3	11.10	24.71	3.89
5	26.37	44.14	8.19
7	38.45	58.03	17.21
10	50.11	71.63	20.41
15	73.71	96.34	39.56
20	77.60	113.55	48.72
25	77.60	116.88	53.72
30	77.60	116.88	53.72

TableS4. Table of emission factor coefficient during diesel fuel used.

**Pollutant type**	**Electric power**	**CO_2**	**NO_X**	**SO_2**	**CO**
—	1.87 (kWh)	3.0959 (kg-/kg)	2.0357 (kg-/kg)	0.0863 (kg-/kg)	1.1756 (kg-/kg)

TableS5. Impact factor potential

**Pollutant type**	**GWP**	**Pollutant type**	**AP**	**Pollutant type**	**EP**	**Pollutant type**	**HTP**
CO_2	1.00	SO_2	1.00	NO_3^-	1.00	CO	1.00
CO	2.00	NO_X	0.70	NO_X	1.35	NO_X	65.00
CH_4	25.00					SO_2	100.00

TableS6. Standardized factors for various types of environmental impacts.

Environmental impact type	normalized factor
GWP	2.37E-14
AP	4.18E-12
EP	6.33E-12
HTP	3.18E-13
